# Construction and analysis of dysregulated lncRNA-associated ceRNA network identified novel lncRNA biomarkers for early diagnosis of human pancreatic cancer

**DOI:** 10.18632/oncotarget.10891

**Published:** 2016-07-28

**Authors:** Meng Zhou, Zhiyong Diao, Xiaolong Yue, Yang Chen, Hengqiang Zhao, Liang Cheng, Jie Sun

**Affiliations:** ^1^ College of Bioinformatics Science and Technology, Harbin Medical University, Harbin, 150081, PR China; ^2^ Department of Plastic Surgery, the First Affiliated Hospital of Harbin Medical University, Harbin, 150001, PR China; ^3^ Medical Oncology Department, Affiliated Tumor Hospital, Harbin Medical University, Harbin, 150001, PR China

**Keywords:** competing endogenous RNAs, ceRNA network, early diagnosis, long non-coding RNAs, pancreatic cancer

## Abstract

It is increasing evidence that ceRNA activity of long non-coding RNAs (lncRNAs) played critical roles in both normal physiology and tumorigenesis. However, functional roles and regulatory mechanisms of lncRNAs as ceRNAs in pancreatic ductal adenocarcinoma (PDAC), and their potential implications for early diagnosis remain unclear. In this study, we performed a genome-wide analysis to investigate potential lncRNA-mediated ceRNA interplay based on “ceRNA hypothesis”. A dysregulated lncRNA-associated ceRNA network (DLCN) was constructed by utilizing sample-matched miRNA, lncRNA and mRNA expression profiles in PDAC and normal samples in combination with miRNA regulatory network. The results of network analysis uncovered seven novel lncRNAs as functional ceRNAs whose aberrant expression will result in the extensive variation in tumorigenic or tumor-suppressive gene expression through DLCN at the post-transcriptional level contributing to PDAC. Therefore, we developed a 7-lncRNA signature (termed LncRisk-7) based on the expression data of seven lncRNAs and SVM algorithm as a novel diagnostic tool to improve early diagnosis of PDAC. The LncRisk-7 achieved high performance in distinguishing PDAC patients from nonmalignant pancreas samples in the discovery cohort and was further confirmed in another two independent validation cohorts. Functional analysis demonstrated that seven lncRNA biomarkers act as ceRNAs involving the regulation of cell death, cell adhesion and cell cycle. This study will help to improve our understanding of the lncRNA-mediated ceRNA regulatory mechanisms in the pathogenesis of PDAC and provide novel lncRNAs as candidate diagnostic biomarkers or potential therapeutic targets.

## INTRODUCTION

Pancreatic cancer (PC) remains the most common cause of cancer-related mortality worldwide [1]. Pancreatic ductal adenocarcinoma (PDAC) is the most frequent subtype of pancreatic cancer, accounting for more than 85% of pancreatic tumor cases [2]. Despite increasing efforts in PDAC, the 5-year survival rate is low at less than 5%, primarily due to late stage diagnosis at advanced stages in a large proportion of patients [1]. To decrease mortality and improve the management of PDAC, diagnostic markers is critical for early detection and risk stratification of PDAC which could aid clinicians to select early tailored treatment. However, the PDAC is a heterogeneous disease in various aspects including clinicopathological, molecular and cellular heterogeneity [3, 4]. Therefore, traditional recognizable clinical and pathological symptoms or signs have limited value in detecting early PDAC. Molecular biomarkers have been proven to be a promising clinical tool for identifying patients' subgroup having early-stage disease.

Over the last ten years, large-scale genome and transcriptome studies have documented large numbers of non-coding RNAs (ncRNAs), including short ncRNAs and long ncRNAs [5, 6]. Long non-coding RNAs (lncRNAs), a major class of ncRNAs with larger than 200 nucleotides in length, have been reported to participate in a wide range of biological processes, including genomic imprinting, transcriptional and post-transcriptional regulation [7–9]. It has been shown that lncRNAs harboring miRNA response elements (MREs) can be regulated by miRNAs, thus acting as competing endogenous RNAs (ceRNAs) to communicate with mRNAs by competing for shared miRNAs [10, 11]. Experimental evidence revealed that dysregulated expressions of key lncRNAs of ceRNA network have greater effects on the miRNA-mediated lncRNA/mRNA ceRNA crosstalk interactions and disrupt bistable states, thus contributing to the initialization and development of cancers [11, 12]. Wang and his colleagues showed that the crosstalk between lncRNA *HULC* and *PRKACB* gene via competitive binding to *miR-372* in a ceRNA-dependent feed-forward loop resulted in highly up- regulated expression of *HULC* in liver cancer [13]. Paci *et al*. built two miRNA-mediated lncRNA-mRNA networks in normal and pathological breast tissue and investigated the potential role of lncRNA as ceRNA in breast cancer [14]. Recent two studies had led to important insights into ceRNA-mediated gene regulation in gastric cancer and ovarian cancer and identified candidate functional cancer-related lncRNAs [15, 16]. Several lncRNAs have been found to be differentially expressed in PC tissues compared to the healthy controls, such as *HOTTIP-005*, *RP11-567G11.1* and *MALAT1* [17, 18], implying their potentials as diagnostic or prognostic biomarkers in PC. A more recent study reported that lncRNA *NUTF2P3-001* function as ceRNAs to communicate with *KRAS* mRNA by competitively binding to *miR-3923*, and the overexpression of *NUTF2P3-001* will unregulated *KRAS* expression by depriving the inhibition of *miR-3923* on *KRAS* leading to the proliferation and invasion of pancreatic cancer cell [19], revealing the functional roles of lncRNA-mediated ceRNA crosstalk in PC for the first time.

In this study, as demonstrated by Chou's 5-step rule [20] to establish a really useful prediction method for a biological system, we first investigated the altered expression patterns of lncRNAs, miRNAs and mRNAs between PDAC patients and normal samples. Then a dysregulated lncRNA-associated ceRNA network (DLCN) was constructed by integrating altered lncRNAs, miRNAs, mRNAs and their co-dysregulated regulatory relationships based on “ceRNA hypothesis”. We uncovered 7 novel lncRNAs as functional ceRNAs with key roles in the pathogenesis of PDAC, and developed a SVM-based 7-lncRNA signature (termed LncRisk-7) which significantly discriminate PDAC tumors from nonmalignant pancreas samples with high performance. The diagnostic values of the LncRisk-7 were further validated in another two independent testing cohorts. With further experimental validation, these novel lncRNAs acting as ceRNAs at the post-transcriptional level may become promising diagnostic biomarkers and therapeutic targets.

## RESULTS

### Identification of differentially expressed mRNAs, miRNAs and lncRNAs in PDAC patients

We first compared the expression profiles of mRNAs, miRNAs and lncRNAs between 25 PDAC samples and 7 nonmalignant pancreas samples from the discovery cohort, and found that 1032 mRNAs, 87 miRNAs and 78 lncRNAs are differentially expressed (Fold change ≥ 1.5 or ≤ 0.67 and FDR-adjusted *p* ≤ 0.1) in PDAC samples compared with nonmalignant pancreas samples using the SAM analysis (Supplementary Table S1). Of these, 632 mRNAs, 47 miRNAs and 52 lncRNAs were over-expressed and 400 mRNAs, 40 miRNAs and 26 lncRNAs were down-expressed in PDAC patients compared with nonmalignant pancreas samples.

### Construction and analysis of dysregulated lncRNA-associated ceRNA network

**Figure 1:**
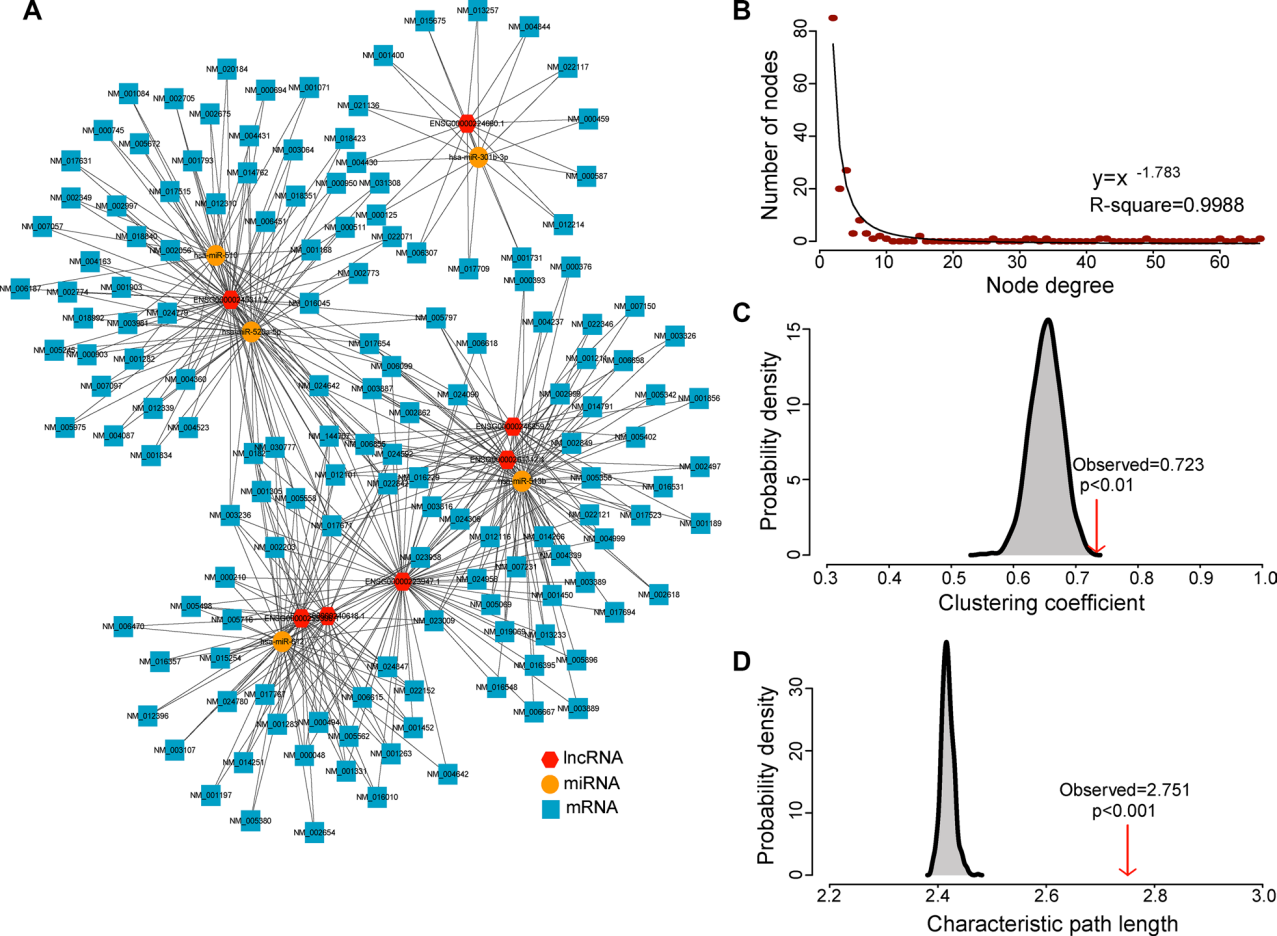
The layout of dysregulated lncRNA-mediated ceRNA network (DLCN) and its structural characteristics (**A**) Global view of the DLCN in pancreatic cancer. The DLCN was comprised of 467 edges among 5 miRNAs, 7 lncRNAs and 150 mRNAs. (**B**) Degree distribution of the DLCN. (**C**) The clustering coefficient of the DLCN is higher than randomization test. The arrow represents the clustering coefficient in the real ceRNA network. (**D**) The characteristic path length of the DLCN is higher than randomization test. The arrow represents the characteristic path length in the real ceRNA network.

The preliminary dysregulated lncRNA-associated ceRNA network (DLCN) was first built by integrating expression profiles and regulatory relationships of mRNAs, miRNAs and lncRNAs of 32 samples in the discovery cohort. As described in the Materials and Methods section, we detected 290 miRNA-mediated lncRNA-mRNA competing triplets among 5 miRNAs, 7 lncRNAs and 150 mRNAs (Supplementary Table S2). These significant competing triplets were then assembled to constitute dysregulated lncRNA-associated ceRNA network for exploring the dynamic changes of ceRNA regulation in PDAC. The resulted DLCN was comprised of 467 edges among 5 miRNAs, 7 lncRNAs and 150 mRNAs (Figure 1A).

**Figure 2:**
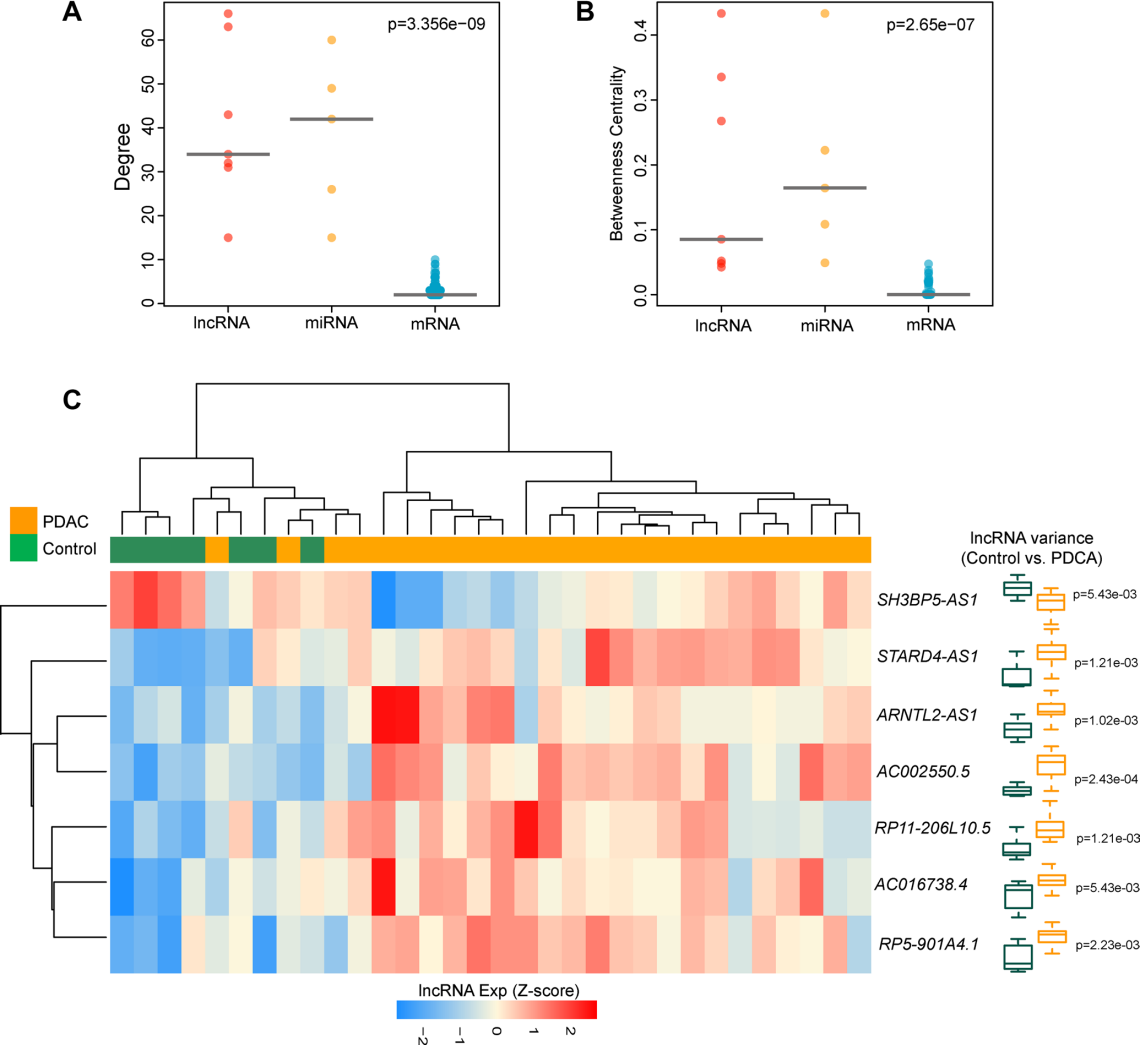
The lncRNA ceRNAs are more critical components compared to mRNA ceRNAs in the DLCN (**A**) The difference of degree among lncRNAs, miRNAs and mRNAs. The lncRNA ceRNAs have significantly higher degrees than mRNA ceRNAs in the DLCN. (**B**) The difference of betweenness centrality among lncRNAs, miRNAs and mRNAs. The lncRNA ceRNAs had a higher betweenness centrality than mRNA ceRNAs in the DLCN. (**C**) The unsupervised hierarchical clustering heatmap of 32 samples based on the expression profiles of 7 lncRNA biomarkers in the discovery cohort.

Next, we performed network analysis to study the structure and organization of the DLCN. The degree distribution of nodes in the DLCN was investigated and the power-law distribution with a slope of −1.783 and R^2^ = 0.9988 was observed (Figure 2B), suggesting that the DLCN displayed scale-free characteristics typical of biological networks. Furthermore, the DLCN showed module characteristics with a significantly higher clustering coefficient than random networks (*p* < 0.01, Figure 2C). However, the characteristic path length of the DLCN is substantially larger than that of random networks (*p* < 0.001) (Figure 1D), implying that the DLCN had reduced global efficiency. The scale-free, module characteristics and reduced global efficiency of the DLCN suggested that dysregulated ceRNA interactions often occurred at a local scale and hubs in the modules tended to be critical in the context of the entire network.

Further comparison analysis showed that there were significant differences in the degree distribution and betweenness centrality among mRNAs, miRNAs and lncRNAs (*p* = 3.356e-09 for degree and *p* = 2.65e- 07 for betweenness centrality, Kruskal-Wallis test) (Figure 2A and 2B). LncRNAs and miRNAs have significantly higher degree and betweenness centrality compared with mRNAs, suggesting that lncRNAs and miRNAs tended to be hub nodes. As showed in Figure 1A, a large proportion of mRNAs (55.3%) communicated with individual lncRNAs, and all lncRNAs acted as ceRNAs to communicate with multiple mRNAs by competing specific shared miRNAs. These results suggested that the aberrant expression of lncRNA ceRNA would result in the extensive variation in gene expression by miRNA-mediated lncRNA-mRNA ceRNA crosstalk interactions, implying that ceRNA function of lncRNAs in the DLCN is of crucial importance in the development of PDAC.

### Identification of potential diagnostic lncRNA signature in PDAC from the discovery cohort

**Figure 3:**
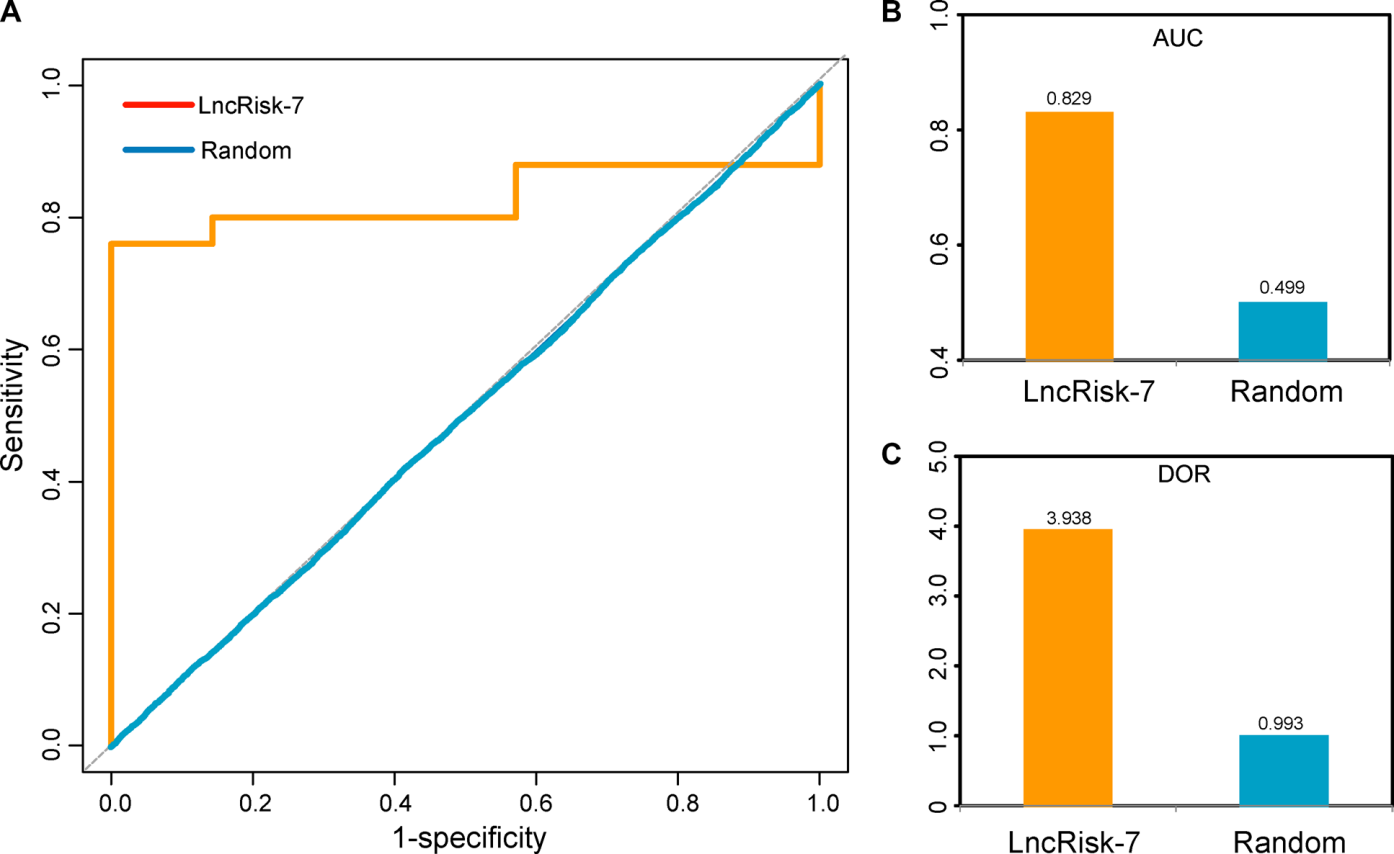
The performance of the LncRisk-7 in distinguishing PDAC patients from nonmalignant pancreas samples in the discovery cohort (**A**) ROC analysis of the sensitivity and specificity of the LncRisk-7. (**B**) The AUC values of the LncRisk-7 based on leave-one-out cross validation. (**C**) The DOR values of the LncRisk-7 based on leave-one-out cross validation.

Based on the above observation, these 7 lncRNAs with ceRNA activity were considered as potential biomarkers associated with PDAC (Table 1). To test whether these 7 lncRNA biomarkers could efficiently distinguish PDAC patients from nonmalignant pancreas samples, we performed unsupervised hierarchical clustering for 32 samples in the discovery cohort according to the expression pattern of 7 lncRNA biomarkers. The results showed that all samples in the discovery cohort were grouped into two distinctive sample clusters (11 samples in Cluster 1 vs. 21 samples in Cluster 2), which were highly correlated with disease status (*p* = 9.804e- 05, Fisher exact test; Figure 2C). As seen in Figure 2C, all nonmalignant pancreas samples were clustered into Cluster 1, and most of PDAC patients (23/25, 84%) were subdivided into Cluster 2. The above results demonstrated that these 7 dysregulated lncRNAs in the DLCN might have a predictive role and could be used as biomarkers in the diagnosis of PDAC. Thus, we integrated these 7 lncRNA biomarkers to construct a 7-lncRNA signature (termed LncRisk-7) by developing an SVM classifier. The performance of the LncRisk-7 in distinguishing PDAC patients from nonmalignant pancreas samples was evaluated in the discovery cohort using the LOOCV procedure, in which 31 samples were used as training set and the remaining one was served as the test sample. Results of discovery cohort showed that the LncRisk-7 was able to correctly classify 24 out of 32 samples, achieving an overall predictive accuracy of 75% with a sensitivity of 84% and a specificity of 57.1%. The discriminatory performance of the LncRisk-7, evaluated by calculating the AUC and DOR, revealed that the AUC was 0.829 (Figure 3A and 3B) and the DOR was 3.938 (Figure 3C), which were significantly higher than those of randomization tests (Figure 3B and 3C). These results demonstrated that the LncRisk-7 had the better predictive performance for discriminating PDAC tumors from nonmalignant pancreas samples.

**Table 1:** The detailed information of dysregulated 7 lncRNAs with ceRNA activity in the DLCN

Ensembl ID	Gene name	Genomic location	Fold change	FDR
ENSG00000224660	SH3BP5-AS1	Chr 3: 15,254,184–15,264,493 (+)	0.42	0.09713
ENSG00000246859	STARD4-AS1	Chr 5: 111,512,226–111,739,726 (+)	2.47	0.09713
ENSG00000245311	ARNTL2-AS1	Chr 12: 27,389,789–27,446,625 (–)	2.78	0.09713
ENSG00000261312	AC002550.5	Chr 16: 19,706,351–19,715,383 (+)	6.63	0.09713
ENSG00000240618	RP11-206L10.5	Chr 1: 759,032–764,925(–)	2.21	0.09713
ENSG00000223947	AC016738.4	Chr 2: 100,993,676–101,002,244(–)	2.67	0.09713
ENSG00000255306	RP5-901A4.1	Chr 11: 68,024,809–68,030,461(–)	3.12	0.09713

### Further validation of the LncRisk-7 with two additional independent PDAC cohorts

**Figure 4:**
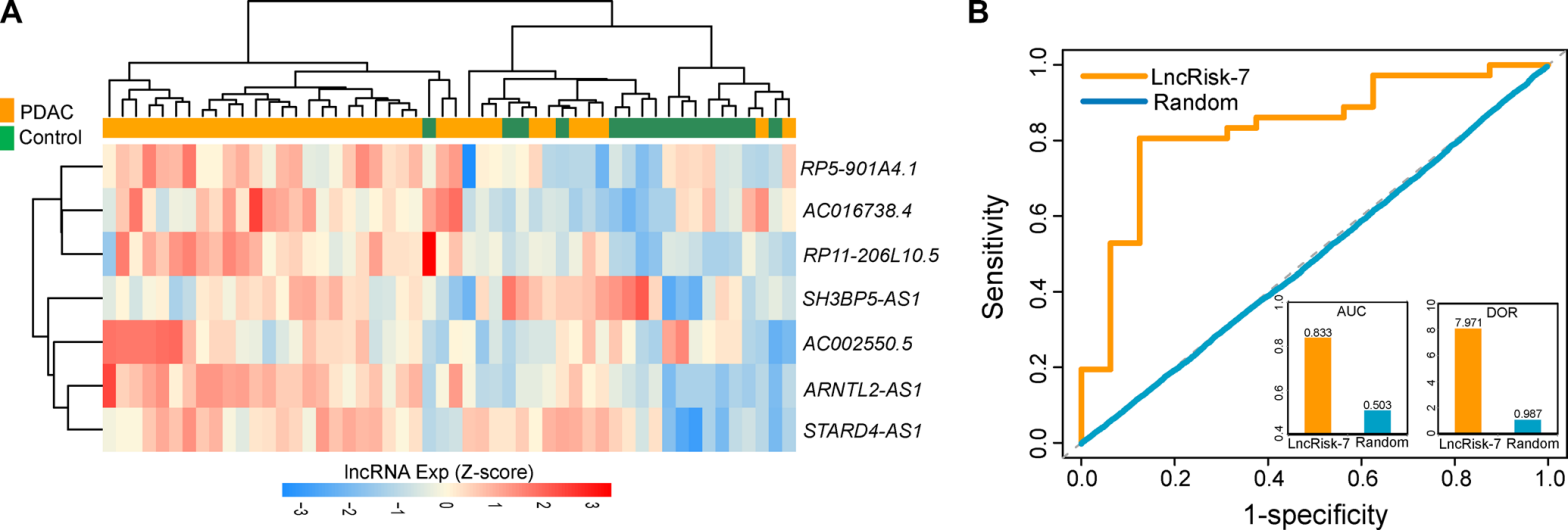
Independent validation of the LncRisk-7 for early diagnosis in another cohort of 52 samples from Pei's study (**A**) Hierarchical clustering heatmap and dendrogram of 52 samples based expression patterns of 7 lncRNA biomarkers. (**B**) The discriminatory performance of the LncRisk-7, evaluated by ROC analysis and calculating the AUC and DOR.

To independently validate the diagnostic power of the LncRisk-7, we first test the discriminatory performance of the LncRisk-7 in an independent cohort of 52 samples from Pei's study [21]. For this, we first performed unsupervised hierarchical clustering analysis based on the expression profiles of 7 lncRNAs and found that 52 samples were grouped into two distinctive clusters based on expression patterns of seven lncRNAs, with significantly different tumor status (*p* = 4.241e- 05, Chi-square test; Figure 4A). Furthermore, the LncRisk-7 efficiently distinguished PDAC patients from nonmalignant pancreas samples in the Pei cohort, achieving 76.9% prediction accuracy with 86.1% sensitivity and 43.8% specificity. The discriminatory power measured by the AUC and DOR was 0.833 and 7.97, respectively (Figure 4B).

**Figure 5:**
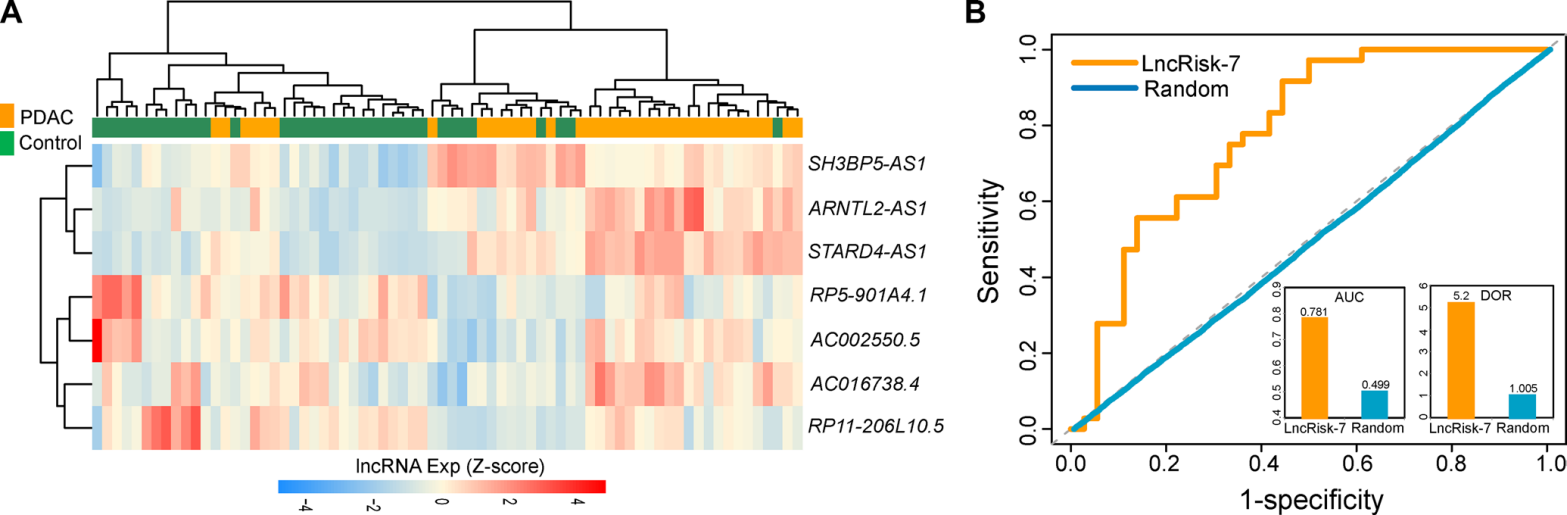
Further confirmation of the LncRisk-7 for early diagnosis in other independent cohort of 72 samples from Badea's study (**A**) Hierarchical clustering heatmap and dendrogram of 72 samples based expression patterns of 7 lncRNA biomarkers. (**B**) The discriminatory performance of the LncRisk-7, evaluated by ROC analysis and calculating the AUC and DOR.

Further validation of the discriminatory power of the LncRisk-7 was conducted using another completely independent cohort of 72 samples from Badea's study [22]. Results with unsupervised hierarchical clustering analysis were similar to those observed in the discovery cohort and Pei cohort above (Figure 5A). Most of PDAC patients (30/36, 83.3%) was clustered into one subgroup and 28 out of 36 (77.8%) normal samples was divided into another subgroup, indicating the significant association between expression patterns of 7 lncRNAs and tumor occurrence (*p* = 7.144e-07, Chi-square test; Figure 5A). Next, we performed classification of PDAC and control samples in the Badea cohort using the LncRisk-7, and found that the LncRisk-7 was able to correctly classify 26 out of 36 PDAC samples and 24 out of 36 control samples, resulting in 69.4 % accuracy, 72.2% sensitivity and 33.3% specificity. The discriminatory power measured by the AUC and DOR was 0.781 and 5.2, respectively (Figure 5B). Taken together, these results with additional independent validation cohorts demonstrated better and reproducible diagnostic performance of the LncRisk-7 in discriminating PDAC tumors from normal samples.

### Functional implication of lncRNA biomarkers

**Figure 6:**
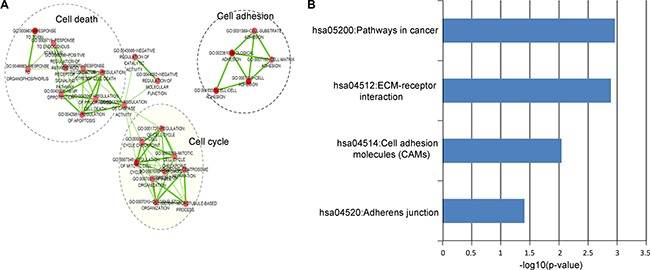
Functional analysis of the diagnostic lncRNAs (**A**) The functional enrichment map of GO terms for mRNAs as ceRNA counterparts of lncRNA biomarkers. Each node represents a GO term and an edge represents the proportion of shared genes between connecting GO terms. (**B**) The enriched KEGG pathways ranked by −log10 (*p*-value).

To investigate the potential functional implication of the LncRisk-7, we performed functional enrichment analysis of GO and KEGG for mRNAs in the DLCN. Results of GO analysis revealed 39 enriched GO terms in the “Biological Process” (GOTERM-BP-FAT) (*p* < 0.05 and Fold Enrichment > 3.0) (Supplementary Table S3), which could be clustered into three functional sub-networks involved in cell death, cell adhesion and cell cycle (Figure 6A). KEGG analysis focusing on the biological pathways showed that these mRNAs as ceRNA interactors of lncRNA biomarkers in the LncRisk-7 were significantly enriched in several pathways involved in pathways in cancer, ECM-receptor interaction, cell adhesion molecules (CMAs) and adherens junction (*p* < 0.05 and Fold Enrichment > 3.0) (Figure 6B and Supplementary Table S3). These enriched biological processes and pathways have been reported to play important roles in PDAC pathogenesis, thus the LncRisk-7 might be involved with.

## DISCUSSION

PDAC is one of the deadliest solid tumors characterized by complex molecular and cellular heterogeneity [4, 23]. During the past years, great efforts have been made to provide novel insights into the molecular mechanisms underlying PDAC, but the focus has been on protein-coding genes or miRNAs [24, 25]. A recently discovered non-coding RNA class, termed lncRNAs, has been widely reported to participate in a wide range of biological processes and their dysregulated expression is associated with many complicated human disease phenotypes including cancers [26–28]. There is increasing evidence that lncRNAs are extensively targeted by miRNAs, and function as ceRNAs. The crosstalk between ceRNAs occurred through competitively binding to shared miRNAs and formed a complex ceRNA-mediated regulatory network contributing to a novel dimension of post-transcriptional gene regulation [10, 11]. It has been shown that lncRNAs are key components of ceRNA-mediated regulatory network and their ceRNA activity has been implicated in both physiological conditions and cancer development [12, 29, 30]. Systematic analysis of ceRNA network has been performed in breast cancer [14, 31], gastric cancer [16], glioblastoma multiforme [32] and ovarian cancer [15]. However, lncRNAs are known to have more developmental stage-, tissue-, organ- and disease-specific expression patterns than protein-coding genes, suggesting that lncRNA-associated ceRNA crosstalk will likely shift under different specific conditions and occur in a disease-specific manner. Therefore, it is critical for studying functional roles and regulatory mechanisms of lncRNA as ceRNAs in the development of PDAC, and investigating their potential implications for early diagnosis of PDAC.

In this study, we obtained genome-wide expression profiles of lncRNAs in PDAC patients and nonmalignant pancreas samples by repurposing three publicly available PDAC-related GEO cohorts and identified 78 differentially expressed lncRNAs, implying that these lncRNAs may be associated with PDAC. In order to explore the ceRNA activity of lncRNAs in PDAC, we used an integrative computational framework to identify 290 dysregulated lncRNA-mediated ceRNA crosstalks through integrating sample-matched expression profiles of lncRNAs, miRNAs and mRNAs with miRNA-target regulatory information and constructed a lncRNA-mediated ceRNA regulatory network. Only 7 of 78 differentially expressed lncRNAs displayed ceRNA activity and communicated with 150 mRNAs by competing for 5 common miRNAs in PDAC. Network analysis showed that PDAC-specific DLCN was a scale-free and small world network as general biological networks, and the topological properties of DLCN were significantly distinguished from random networks, including high clustering coefficient and characteristic path length. We further examined the associations between mRNAs ceRNAs in the DLCN and cancers, and found that 150 mRNA ceRNAs in the DLCN were significantly enriched in the cancer class from The Genetic Association Database [33] (GAD, https://geneticassociationdb.nih.gov/) (*p* = 5.0e-03), in which 40 mRNAs are known to be related to cancers recorded in GAD (Supplementary Table S4). Previous studies have suggested that hub nodes, characterized by their high degree of connectivity to other nodes, play critical roles in a network and tend to be essential in network organization [34, 35]. In the DLCN, lncRNAs were observed to be topological key nodes whose degree and betweenness centrality are significantly higher than mRNA ceRNAs, indicating that ceRNA activity of lncRNAs has profound implications for PDAC and the dysregulated expression of lncRNA ceRNAs will result in the extensive variation in tumorigenic or tumor-suppressive gene expression through lncRNA-mediated ceRNA regulatory network at the post-transcriptional level contributing to PDAC.

With increasing attention to the roles of lncRNAs as oncogenes and tumor suppressors in cancers, lncRNAs have exhibited superior potential as diagnostic and prognostic biomarkers than protein-coding genes owing to the more closely association between lncRNA expression and their functions [36, 37]. Recent studies have reported several lncRNA-focus signatures to improve prognosis prediction for some malignant tumors including breast cancer, lung cancer, colorectal cancer and so on [38–45]. However, the diagnostic role of lncRNAs in PDAC has not been investigated. Introducing graphic methods into biological systems can provide an intuitive vision and useful insights. It is particularly helpful to deal with complicated biological systems as demonstrated in recent some studies [46–48]. To explore whether 7 lncRNAs with ceRNA activity uncovered in the DLCN could become suitable diagnostic biomarkers for PDAC, we first performed unsupervised hierarchical clustering analysis and found that expression patterns of 7 lncRNAs could discriminate effectively between PDAC tumors and nonmalignant pancreas samples in the discovery cohort and two independent validation cohorts. In statistical prediction, the following three cross-validation methods are often used to examine a predictor for its effectiveness in practical application: independent dataset test, subsampling test, and jackknife test [49]. We adopted the leave one out cross-validation in this study as done by many investigators with SVM as the prediction engine and chose independent dataset test method to examine the accuracy of 7 lncRNAs in dignostic precdition. Therefore, we developed a 7-lncRNA signature based on the expression data of 7 lncRNAs and SVM algorithm as a novel diagnostic tool to improve early diagnosis of PDAC. The 7-lncRNA signature achieved a high performance in distinguishing PDAC patients from nonmalignant pancreas samples in the discovery cohort and was further confirmed in another two independent validation cohorts. These results demonstrated that the 7-lncRNA signature had the robust discriminatory power and may become a reliable and powerful predictor for early diagnosis of patients with PDAC.

As functional characterization of lncRNA is still in its infancy, only very few lncRNAs have been well functionally annotated until now. The ceRNA activity of these 7 lncRNAs was reported to be associated with PDAC for the first time and their function is unknown. It is increasing evidence that ceRNA crosstalk has become a powerful approach to infer functional roles of lncRNA with unknown function based on their ceRNA activity [27, 28, 50–52]. Therefore, as a preliminary exploration for the potential functional implication of the LncRisk-7, we used ‘guilt by association’ principle to investigate biological processes and pathways regulated by the dysregulation of lncRNAs biomarkers and associated ceRNA crosstalk by functional enrichment analysis for mRNAs in the DLCN. We found that mRNAs as ceRNA counterparts of lncRNA biomarkers were involved in three GO biological processes (cell death, cell adhesion and cell cycle) and four KEGG pathways (pathways in cancer, ECM-receptor interaction, cell adhesion molecules (CMAs) and adherens junction). Three GO biological processes(cell death, cell adhesion and cell cycle) are well known to be associated with the hallmarks of cancer [53]. The extracellular matrix (ECM) not only provided mechanical and structural support but also played important roles in the development processes [54]. The dysregulated expression of ECM receptors contributing to the alterations of ECM formation and composition has been implicated in PDAC [55, 56]. Therefore, it is a plausible inference that the seven lncRNA biomarkers act as ceRNAs involving the regulation of cell death, cell adhesion and cell cycle. The dysregulation of these lncRNA ceRNAs and the resultant perturbation in lncRNA-mediated ceRNA network will have important effects in cancer-related biological processes contributing to tumorigenesis and progress of PDAC.

In summary, we performed a genome-wide analysis to investigate potential lncRNA-mediated ceRNA interplay by utilizing sample-matched miRNA, lncRNA and mRNA expression profiles in PDAC patients and normal samples in combination with miRNA regulatory network based on “ceRNA hypothesis”. Then a PDAC-specific dysregulated lncRNA-mediated ceRNA network was constructed, which for the first time enables an overall view and analysis of lncRNA-associated ceRNA-mediated gene regulation in the development of PDAC on a system-wide level. This study will help to improve our understanding of lncRNA-mediated ceRNA regulatory mechanisms in the pathogenesis of PDAC and provide novel lncRNAs as candidate diagnostic biomarkers or potential therapeutic targets.

## MATERIALS AND METHODS

### Patient datasets

Three independent, nonoverlapping PDAC patient cohorts were used in this study. The initial discovery cohort of 25 PDAC samples and 7 nonmalignant pancreas samples with whole genome gene expression profiles and miRNA expression profiles were retrieved from Donahue' study [63] and were used to identify dysregulated lncRNA-mediated ceRNA interplay in PDAC. Another two PDAC patient cohorts composed of 52 samples (36 tumor samples and 16 normal samples, denoted by “ Pei cohort”) and 72 samples (36 tumor samples and 36 normal samples, denoted by “ Badea cohort”) only with whole genome gene expression profiles were collected from Pei's study [21] and Badea's study [22], and were used as validation cohorts to test the diagnostic power of lncRNA biomarkers.

### Acquisition and analysis of expression profiles

The sample-matched whole genome gene expression data and miRNA expression profiles data of 32 samples in the discovery cohort and the whole genome gene expression data of 52 samples in the Pei cohort and 72 samples in the Badea cohort were obtained from the public available GEO database (the GEO accession number is GSE32688, GSE16515 and GSE15471). Raw gene expression data profiled from Affymetrix Human Genome U133 Plus 2.0 Array (HG-U133_Plus_2.0) in the three patient cohorts were processed and normalized using the Robust Multichip Average (RMA) algorithm for background adjustment [64] and log-transformed (base 2). miRNA expression data produced by the Exiqon miRNA arrays (miRCURY LNA microRNA Array v.11.0 -hsa, mmu & rno) were adjusted and normalized by variance stabilizing transformation [63]. lncRNA expression data were obtained by repurposing the probes in the HG-U133_Plus_2.0 array to lncRNAs based on the annotation from the GENCODE project (http://www.gencodegenes.org, release 22) as previously described [36, 41, 65, 66]. Finally, expression data of 11005 protein-coding genes (PCGs), 826 miRNAs and 2330 lncRNAs were retained for further analysis.

Differential expression analysis of mRNAs, miRNAs and lncRNAs between PDAC samples and normal samples was carried out using the significance analysis of microarrays (SAM) method [67]. Unsupervised hierarchical clustering was used to investigate the expression pattern between PDAC samples and normal samples, and the Chi-square test or Fisher exact test was used to analyze the correlations between tumor status and lncRNA biomarkers.

### MiRNA-mRNA and miRNA-lncRNA interaction data

The experimentally validated miRNA-mRNA interaction data was collected and integrated from TarBase (version 6.0) [68], miRTarBase (version 4.5) [69] and miRecords [70], including 37659 interactions between 402 miRNAs and 12360 PCGs. The putative interactions of miRNA-lncRNA were downloaded from lnCeDB database [71], including 1562845 interactions between 1394 miRNA and 28364 lncRNAs.

### Construction and analysis of dysregulated lncRNA-associated ceRNA network

The dysregulated lncRNA-associated ceRNA network (DLCN) in PDAC was constructed based on “ceRNA hypothesis” as follows: Firstly, expression correlation between differentially expressed mRNAs and differentially expressed lncRNAs was evaluated using Pearson correlation coefficient (PCC) from matched mRNA and lncRNA expression profiles data. Those dysregulated lncRNA-mRNA pairs with PCC > 0.5 and *p* < 0.05 were selected as co- dysregulated lncRNA-mRNA pairs. Then, the Pearson correlation coefficient between differentially expressed miRNAs and differentially expressed mRNAs, and between differentially expressed miRNAs and differentially expressed lncRNAs was computed from paired miRNA, mRNA and lncRNA expression profile data. For a given co-dysregulated lncRNA-mRNA pair, both mRNAs and lncRNAs in this pair are targeted and co-expressed negatively with a certain common miRNA, this miRNA-mRNA-lncRNA was identified as dysregulated competing triplets (DCTs). Finally, a DLCN was built for PDAC by assembling all DCTs identified above.

The network characteristics of DLCN, including degree, characteristic path length (CPL), clustering coefficient (CC) and betweenness centrality were analyzed.

### Development of lncRNA-based signature in PDAC diagnosis

For classification of PDAC vs. normal samples, lncRNA biomarkers were integrated to form a lncRNA-focus signature using support vector machine (SVM) with the sigmoid kernel. An unbiased performance estimate in identifying PDAC patients was carried out using leave one out cross-validation (LOOCV). Diagnostic ability of the lncRNA-focus prediction model was evaluated by obtaining the area under a receiver operating characteristic (ROC) curve (AUC) and diagnostic odds ratio (DOR). The ROC curve was produced by plotting true positive rates (sensitivity) against false positive rates (1-specificity). The DORs were calculated as follows:

$$DOR=\frac{Sensitivity\times Specificity}{\left(1-Sensitivity)\times (1-Specificity\right)}$$

The permutation *p*-value of AUC and DOR was obtained from 1,000 randomization tests by randomizing lncRNA expression data for testing the null hypothesis.

### Functional analysis of lncRNA biomarkers

Functional enrichment analysis of Gene Ontology (GO) and Kyoto encyclopedia of genes and genomes (KEGG) for mRNAs in the DLCN was performed to infer potential biological processes and pathways of lncRNA biomarkers using DAVID Bioinformatics Tool (version 6.7) [72] limited to GO terms in the “Biological Process”(GOTERM-BP-FAT) and KEGG pathway categories. The biological processes and pathways with *p*-value of < 0.05 and an enrichment score of > 3.0 using the whole human genome as background were considered as significant functional categories which were organized into an interaction network with similar functions using the Enrichment Map plugin in Cytoscape environment [73].

## SUPPLEMENTARY MATERIALS FIGURES AND TABLES










