# Supplementary material for: Construction and analysis of dysregulated lncRNA-associated ceRNA network identified novel lncRNA biomarkers for early diagnosis of human pancreatic cancer
Source: Oncotarget. 2016 Jul 28;7(35):56383–94. doi: 10.18632/oncotarget.10891 (PMC5302921; doi:10.18632/oncotarget.10891)
Supplement: Supplementary file 1 [file oncotarget-07-56383-s001.pdf]

Construction and analysis of dysregulated lncRNA-associated ceRNA network identified novel lncRNA biomarkers for early diagnosis of human pancreatic cancer

Supplementary Materials

Supplementary Table S1: The differentially expressed lncRNAs, miRNAs and mRNAs between 25 PDAC samples and 7 nonmalignant pancreas samples from the discovery cohort using SAM analysis with an adjusted *p*-value < 0.01 after Bonferroni correction. See Supplementary_Table_S1

Supplementary Table S2: Detailed information of 290 miRNA-mediated lncRNA-mRNA competing triplets among 5 miRNAs, 7 lncRNAs and 150 mRNAs in PDCA. See Supplementary_Table_S2

Supplementary Table S3: Significantly enriched GO terms and KEGG pathways. See Supplementary_Table_S3

Supplementary Table S4: Detailed information of mRNA ceRNAs in the DLCN enriched in the cancer class from The Genetic Association Database. See Supplementary_Table_S4
